# Clinical Remission Predictors in Non-Colonized Bronchiectasis and Severe Asthma with Type 2-Targeted Biologic Therapy: A Retrospective Real-Life Pilot Study

**DOI:** 10.3390/jcm13216309

**Published:** 2024-10-22

**Authors:** Vitaliano Nicola Quaranta, Andrea Portacci, Francesca Montagnolo, Silvano Dragonieri, Ilaria Iorillo, Ernesto Lulaj, Leonardo Maselli, Enrico Buonamico, Giovanna Elisiana Carpagnano

**Affiliations:** Respiratory Diseases, University of Bari, 70121 Bari, Italy; vitalianonicola.quaranta@asl.bari.it (V.N.Q.); andrea.portacci@uniba.it (A.P.); francescamontagnolo17@gmail.com (F.M.); iorillo.ilaria@gmail.com (I.I.); ernesto.lulaj@gmail.com (E.L.); l.maselli7@studenti.uniba.it (L.M.); enricobuonamico@gmail.com (E.B.); elisiana.carpagnano@uniba.it (G.E.C.)

**Keywords:** severe asthma, non-cystic fibrosis bronchiectasis, non-colonized patients

## Abstract

**Background/Objective:** Patients with severe asthma (SA) and non-cystic fibrosis bronchiectasis (BE) without microbiological colonization represent a unique and understudied population. Type 2-targeted biologic therapies have emerged as a promising treatment for these patients. However, predictive factors for achieving clinical remission remain unclear. This study aims to identify the predictive factors for achieving clinical remission in patients with severe asthma and non-colonized bronchiectasis undergoing type 2-targeted biologic therapies. **Methods:** A retrospective longitudinal analysis was conducted on 14 patients with severe asthma and non-cystic fibrosis bronchiectasis without microbiological colonization. Clinical remission was assessed at baseline (T0) and after 12 months (T1) of biologic therapy. Clinical remission was defined according to the Severe Asthma Network Italy (SANI) criteria, including the absence of oral corticosteroid use, no asthma-related symptoms, stable lung function, and no exacerbations. Logistic regression was performed to identify predictors of remission. ROC curves were constructed to evaluate the predictive accuracy of lung function parameters, specifically FEV1 and FVC. **Results:** After 12 months of biologic therapy, 28.6% of patients (*n* = 4) achieved clinical remission. The mean FEV1 percentage at baseline was significantly higher in the remission group (92.25 ± 15.64%) compared to the non-remission group (65.10 ± 23.36%, *p* = 0.034). Logistic regression analysis identified baseline FEV1 as a significant predictor of remission (OR = 1.008, *p* = 0.050). ROC curve analysis revealed that an FEV1 cutoff of 72.5% had a sensitivity of 100% and a specificity of 70% (AUC = 0.900, *p* = 0.024) for predicting clinical remission. **Conclusions:** FEV1 is a crucial predictor of clinical remission in patients with severe asthma and non-colonized bronchiectasis treated with type 2-targeted biologic therapies. An FEV1 threshold of 72.5% can guide clinicians in identifying patients most likely to achieve remission. These findings underline the importance of preserving lung function to optimize therapeutic outcomes in this complex population.

## 1. Introduction

**Figure 1:**
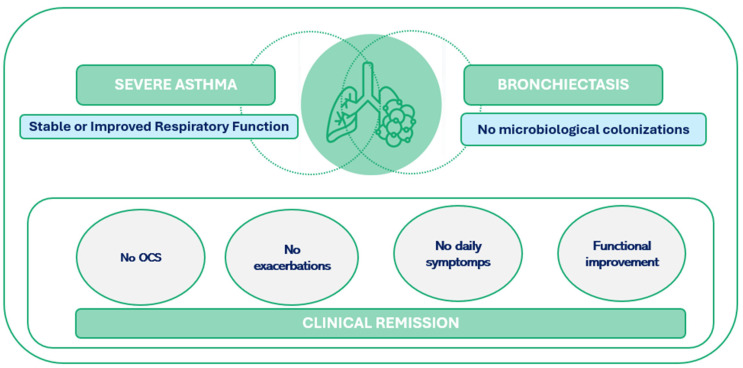
Respiratory function in non-colonized bronchiectasis patients with severe asthma undergoing biologic therapy.

Severe asthma is a complex condition characterized by persistent and intense symptoms that persist despite the use of high-dose inhaled corticosteroids and other controller medications [1]. The introduction of biological therapies has revolutionized the management of severe type 2 (T2) asthma [1], providing significant improvements and enabling many patients to achieve clinical remission [2]. Clinical remission is defined by the absence of oral corticosteroid use, a lack of symptoms and exacerbations, and stable pulmonary function. However, achieving this remission, whether complete or partial, remains a significant challenge, especially in the presence of comorbidities [2] (Figure 1).

Comorbidities complicate asthma prognosis, making it more challenging to control the disease and potentially worsening clinical outcomes. Among these, bronchiectasis is particularly problematic due to its association with recurrent exacerbations and airway damage, which negatively impact prognosis and complicate treatment strategies [3].

The coexistence of bronchiectasis and severe eosinophilic asthma (SEA) represents a distinct phenotype, affecting a considerable portion of severe asthma patients, with prevalence ranging from 24% to 40% [4,5]. This phenotype is frequently associated with older age, late-onset asthma, irreversible airflow obstruction, and frequent exacerbations. The shared inflammatory mechanisms, particularly type 2 (T2) inflammation, not only intensify asthma severity but also contribute to the progression of bronchiectasis, further complicating management.

Patients with both asthma and bronchiectasis require specialized care and close monitoring. Current guidelines stress the importance of addressing comorbidities like bronchiectasis to optimize severe asthma management [1]. Biologic therapies have proven effective in reducing exacerbations, minimizing corticosteroid use, and improving lung function and quality of life, even in the presence of bronchiectasis [2,6]. Recent studies have underscored the effectiveness of Benralizumab [7] and Mepolizumab [4,8] in achieving the ambitious goal of clinical remission in patients with both severe eosinophilic asthma (SEA) and bronchiectasis (BE).

However, chronic colonization can lead to an increase in neutrophilic exacerbations in bronchiectasis [3], potentially impacting the clinical remission of severe asthma. To date, no studies have investigated the role of type 2-targeted biologic therapy in achieving clinical remission in patients with severe asthma and bronchiectasis who do not have any microbiological colonization.

The main objective is to understand the predictive factors of clinical remission in patients with severe asthma (SA) and non-allergic severe bronchial asthma who have non-cystic fibrosis (CF) bronchiectasis and are not colonized by microorganisms after initiating type 2-targeted biologic therapies.

## 2. Materials and Methods

### 2.1. Patients

Our group of patients comprised 14 individuals over 18 years old with severe asthma, as defined by the Global Initiative for Asthma (GINA) [1] and the European Respiratory Society/American Thoracic Society (ERS/ATS) guidelines [9]. All participants also had bronchiectasis, defined as the presence of both permanent bronchial dilatation on CT scans confirmed through chest computed tomography (CT) imaging [3]. The mean age of the population was 58 ± 14.21 (35.7% female). The mean duration of asthma disease was 21.85 ± 17 years.

Inclusion criteria were the presence of patients over 18 years of age with severe asthma with eligibility criteria for biological therapy [1], T2-high inflammation, and the simultaneous presence of bronchiectasis without microbiological colonization.

Exclusion criteria were patients under 18, those unable to perform respiratory function tests, non-compliance with therapy, or having chronic obstructive pulmonary disease (COPD). Individuals with other lung conditions like cystic fibrosis (CF), CF-related bronchiectasis, allergic bronchopulmonary aspergillosis (ABPA), positive tests for Aspergillus Fumigatus, pulmonary mycobacteriosis, previous pulmonary tuberculosis, or pulmonary fibrosis were also excluded.

### 2.2. Study Design

**Figure 2:**
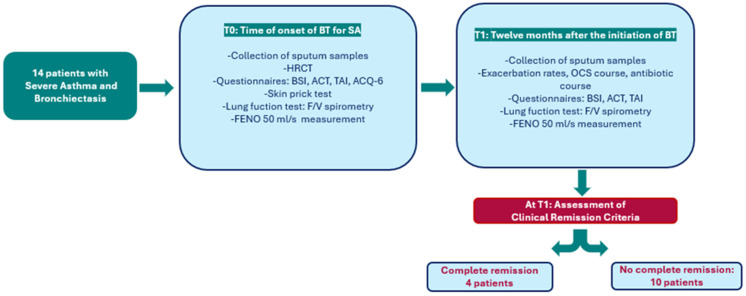
Flowchart of the study. Abbreviations: T0, baseline; T1, 12-month follow-up; BT, biological therapy; HRCT, high-resolution computed tomography; BSI, Bronchiectasis Severity Index; ACT, Asthma Control Test; TAI, Test of Adherence to Inhaler; ACQ-6, Asthma Control Questionnaire; F/V, Flow/Volume; FENO, Fractional Exhaled Nitric Oxide; OCS, Oral Corticosteroids.

We performed a retrospective longitudinal analysis involving 14 patients affected with both severe asthma and bronchiectasis and followed by Severe Asthma Center of the Pulmonology Departments at the University Hospital of Bari. We defined the following time-points:

- T0 time: time of onset of observational study, when biological therapy (BT) for SA was prescribed according to current guidelines [1].
- T1 time refers to 12 months after the initiation of BT, at which point the achievement of a composite endpoint for complete clinical remission of asthma was evaluated. According to Canonica et al. [10], to be considered in complete clinical remission, a patient with severe asthma must satisfy the following criteria: discontinuation of oral corticosteroids (OCS), no asthma-related symptoms, absence of exacerbations or acute attacks, and stable lung function. The term “stable lung function” refers to maintaining forced expiratory volume in 1 s (FEV1) within a variation range of ±5% to ±10% of baseline values over a 12-month period, without significant exacerbations or the need for systemic corticosteroids, in accordance with the criteria for clinical remission in severe asthma. These criteria were established through a Delphi consensus by experts in the Severe Asthma Network Italy (SANI) and serve as a practical tool for assessing treatment efficacy and guiding therapy management in severe asthma patients (Figure 2).

Asthma exacerbations were defined as a worsening of the condition, requiring emergency care, hospitalization, or the use of oral corticosteroids (OCS) for three or more days or a 50% or greater increase in the daily OCS dose. Exacerbations treated with corticosteroid cycles less than seven days apart were considered part of the same exacerbation. Patients were classified as exacerbation-free if they did not experience any asthma exacerbations as per the established guidelines [11].

Chronic mucus hypersecretion (CMH) was defined as the presence of a cough and excessive mucus production in the airways on most days of the week for at least three months per year, occurring over at least two consecutive years [12]. Bronchiectasis exacerbations are characterized by a worsening of daily respiratory symptoms, including increased cough, sputum production, malaise, fatigue, and breathlessness [3].

This study was carried out following the principles of the Declaration of Helsinki, approved by a local Institutional Ethics Committee (Ethical Committee number: 6313, Approval date: 4 March 2020), and all subjects provided informed consent for study participation.

### 2.3. Microbiology Testing and Chronic Colonization Assessment

Sputum samples were collected from patients and analyzed for bacterial, fungal, and mycobacterial cultures to assess chronic colonization. Samples were taken at least twice over a year, with a minimum three-month interval between collections [13]. Patients unable to produce sputum due to a dry cough were considered free of chronic infection for analysis. Individuals with cystic fibrosis or traction bronchiectasis were excluded from the study.

### 2.4. Detection and Assessment of Bronchiectasis

All enrolled patients underwent HRCT scans of the lungs within three months prior to beginning biological therapy. The scans, with slices between 0.5 and 1.5 mm, were used to diagnose bronchiectasis (BE). This diagnosis was made by an expert radiologist based on specific features, including the absence of bronchial tapering, visible bronchi near the costal pleura, and a broncho-arterial ratio greater than 1:1, often indicated by the signet-ring sign [14].

The Bronchiectasis Severity Index (BSI) is a multidimensional scoring system used to assess the severity of bronchiectasis (BE) in patients with severe eosinophilic asthma (SEA) and BE. The BSI incorporates various clinical, radiological, and microbiological factors, including age, Body Mass Index (BMI), predicted %FEV1, hospitalization history over the past two years, the number of exacerbations in the previous year, the modified Medical Research Council (mMRC) dyspnoea score, the extent of radiological involvement (with ≥3 lobes or cystic BE), and the presence of specific pathogens such as Pseudomonas aeruginosa or other organisms. The BSI score ranges from 0 to 26, categorizing BE as mild (0–4 points), moderate (5–8 points), or severe (≥9 points) [15]. We used the FACED score, a clinical assessment tool that evaluates the severity of bronchiectasis by considering five key factors: FEV1 (forced expiratory volume in 1 s), age, chronic Pseudomonas aeruginosa bronchial infection, radiological extension, and dyspnea [16].

We describe the shapes of bronchiectasis as follows: Tubular (or cylindrical), varicose (or Ovalar), and saccular (or cystic), with saccular bronchiectasis being the most severe form [17].

We also measured inflammatory markers, including Fractional Exhaled Nitric Oxide (FeNO) levels and Blood Eosinophil Count (BEC). FeNO was assessed at flow rates of 50 mL/s using the FeNO+ analyzer by Medisoft-MGCD (Saint Paul, MN, USA), adhering to both manufacturer instructions and ERS guidelines [18].

### 2.5. Asthma-Related Questionnaire

Asthma-related questionnaires were administered, including the Asthma Control Test (ACT) [19] and the Asthma Control Questionnaire-6 (ACQ-6). We assessed adherence to prescribed inhalation therapy using the Test of Adherence to Inhalers (TAI) [20].

### 2.6. Skin Prick Test

The skin prick test (SPT) was performed for a panel of inhalant allergens as previously described for common aeroallergens (Lofarma, Milan, Italy). It was considered positive when eliciting a wheal diameter ≥ 3 mm using negative (saline) and positive (histamine 10 mg/mL) controls for interpretation [21].

### 2.7. Lung Function

In accordance with the guidelines [22], FEV1, FVC, and RV were measured using a spirometer with body plethysmography (Jaeger, Essen, Germany). Respiratory tests were performed by experienced technicians under the supervision of a pneumologist. We measured and recorded the forced vital capacity (FVC) and the forced expiratory volume during the first second of the forced breath (FEV1) from the F/V spirometry and the residual volume (RV) from the plethysmography. The best of three reproducible measurements was selected and expressed as a percentage of the predicted value.

### 2.8. FeNO Measurement

FeNO was measured using an electrochemical analyzer (HypairFeNO Medisoft Exp’air, 2010) according to ATS-ERS recommendations for the online measurement of FeNO in adults. FeNO measurement was performed according to guidelines. The measurement range was 0–600 ppb. Exhaled NO (FeNO) was measured using a restricted breathing technique that employed expiratory resistance and positive mouth pressure to close the veil and exclude nasal NO and a constant expiratory flow of 50 mL/s. Repeated exhalations were performed until three plateaus agreed within 5% of the difference between observations [18].

### 2.9. Time Points and Data Collection

The study followed a retrospective, longitudinal design with two primary time points: T0 and T1.

T0 (Baseline): At the initiation of the study, which coincided with the prescription of biological therapy (BT) for severe asthma (SA), a comprehensive set of clinical and functional parameters were collected. This included lung function tests, such as forced expiratory volume in 1 s (FEV1) and forced vital capacity (FVC), which were measured using spirometry in accordance with ATS/ERS guidelines. Baseline assessments also included the number of asthma exacerbations, emergency Room visits, antibiotic use, and oral corticosteroid (OCS) cycles in the previous year. The Asthma Control Test (ACT), Asthma Control Questionnaire-6 (ACQ-6), and Test of Adherence to Inhalers (TAI) were administered to evaluate asthma control and treatment adherence. Additionally, patients underwent microbiological testing on sputum samples, high-resolution computed tomography (HRCT) of the lungs to confirm bronchiectasis, and skin prick tests (SPT) for common aeroallergens.

T1 (12-Month Follow-Up): Twelve months after the initiation of BT, the same parameters were reassessed to evaluate the achievement of complete clinical remission. This included repeated lung function tests, a review of exacerbation rates, OCS usage, and antibiotic use. The ACT, ACQ-6, and TAI scores were also reevaluated. Further, the impact of bronchiectasis on remission was assessed using the Bronchiectasis Severity Index (BSI) and FACED score. The remission criteria were defined according to the Severe Asthma Network Italy (SANI) guidelines, which required the absence of OCS use, no asthma-related symptoms, no exacerbations, and stable lung function.

### 2.10. Comorbidities and Other Parameters Collected

The study also involved the collection of various comorbidities and inflammatory markers at both T0 and T1.

Comorbidities: The presence of chronic rhinosinusitis, nasal polyposis, gastroesophageal reflux disease (GERD), obstructive sleep apnea syndrome (OSAS), depressive/anxious syndrome, and other conditions like vocal cord dysfunction were recorded. The Charlson Comorbidity Index was used to quantify the overall burden of comorbidities.

Inflammatory Markers: Blood eosinophil counts (BECs), serum IgE levels, and Fractional Exhaled Nitric Oxide (FeNO) were measured to assess systemic and airway inflammation. These markers were monitored at both T0 and T1 to correlate with asthma control and the likelihood of achieving clinical remission.

## 3. Statistical Analysis

The Kolmogorov–Smirnov test was used to evaluate the normal distribution of data. Continuous variables that had a non-parametric distribution expressed as a median and interquartile range 25–75 (IQ 25–75). The continuous variable that had parametric distribution was expressed as mean Standard Deviation (SD). The population was divided into two subgroups based on the achievement of complete remission outcome: those who achieved complete clinical remission (*n* = 4) and the others (*n* = 10). Categorical values were analyzed using the chi-square test or Fisher’s exact test as appropriate and were reported as *n* (%). Continuous variables were compared Mann–Whitney U test for non-normally distributed data and The Independent Samples t Test for normally distributed data.

Univariate binomial logistic regression analyses were performed to define the probability of having complete remission.

To detect the accuracy in predicting complete remission starting from the parameters found to be significant from the comparison analysis between populations, ROC curves were constructed by plotting the true positive rate (sensitivity) against the false positive rate (1-specificity) across various threshold levels. The area under the curve (AUC) was calculated to summarize the overall diagnostic performance of the test. An AUC of 0.5 indicates no discriminative ability, while an AUC closer to 1.0 reflects excellent test performance. The Youden Index was used to determine the optimal threshold for a diagnostic test by maximizing the sum of sensitivity and specificity. The sensitivity, specificity, positive predictive value, and negative predictive value of the cutoff in predicting complete remission were calculated.

Significance values were assumed for *p* < 0.050. All statistical analyses were performed using SPSS for Windows 25.0 (SPSS, Chicago, IL, USA).

## 4. Results

### 4.1. Population Description

The study consisted of 14 patients diagnosed with severe asthma and bronchiectasis, with a mean age of 58 ± 14.21 years. Among these patients, 35.7% were female. The average duration of asthma was 21.85 ± 17 years, and the mean age at diagnosis was 36.35 ± 23.11 years. The majority of patients were non-smokers (64.3%), and 64.3% had a history of allergies. No patients in the study were affected by the following conditions: eosinophilic granulomatosis with polyangiitis (EGPA), eosinophilic pneumonia, hypereosinophilic syndrome, urticaria, vocal cord dysfunction, obstructive sleep apnea syndrome (OSAS).

Baseline clinical assessments showed an average FEV1 percentage of 72.85 ± 24.42% and an FVC percentage of 86.14 ± 18.94%. The mean eosinophil count was 465.21 ± 252.92 cells/µL, and the median IgE level was 258.88 ± 566.84 IU/mL. Patients had a mean ACT score of 19.5 (range: 18–22) and an ACQ-6 score of 1.95 (range: 1.6–4.5). The Bronchiectasis Severity Index (BSI) varied among patients, with a median score of 6.5, categorizing most patients as having moderate bronchiectasis severity. Regarding biological therapies, our group of patients was treated with different agents, including Omalizumab (*n* = 3), Mepolizumab (*n* = 2), Benralizumab (*n* = 6), and Dupilumab (*n* = 3) (Table 1).

### 4.2. Comparison Between Groups

Patients were categorized into two groups based on their achievement of complete clinical remission at the 12-month follow-up. Four patients achieved remission, while 10 did not. Notably, those who reached remission exhibited significantly higher lung function, with an FEV1 percentage of 92.25 ± 15.64% compared to 65.10 ± 23.36% in the non-remission group (*p* = 0.034). Similarly, the FVC percentage was markedly higher in the remission group (101.75 ± 8.18%) compared to the non-remission group (79.90 ± 18.56%, *p* = 0.010). These differences highlight the association between better baseline lung function and the likelihood of achieving remission. Additionally, the remission group tended to have lower Bronchiectasis Severity Index scores, although this difference was not statistically significant (Table 1).

### 4.3. Prediction Analysis

Univariate binomial logistic regression analysis was performed to identify predictors of complete clinical remission. The analysis demonstrated that a higher baseline FEV1 percentage was a significant predictor of achieving remission (OR = 1.008, *p* = 0.050). Other factors, such as age, BMI, smoking status, and disease duration, did not show a significant association with remission outcomes. Although several variables were assessed, lung function (particularly FEV1) emerged as a key determinant of remission likelihood, underscoring the importance of maintaining or improving pulmonary function in severe asthma management (Table 2).

### 4.4. ROC Curve Analysis

**Figure 3:**
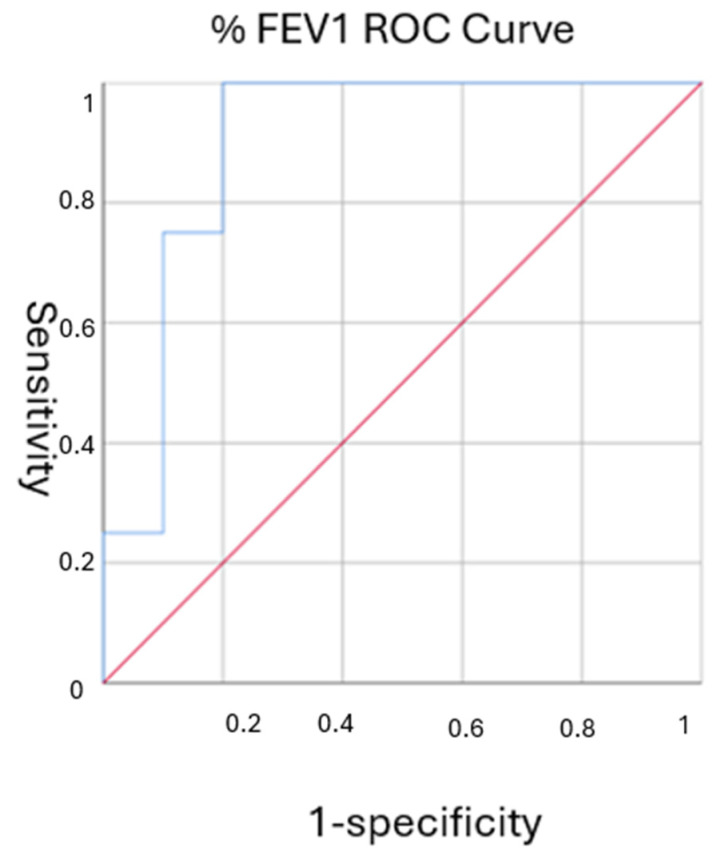
%FEV1 ROC Curve: Predictive Accuracy of % FEV1 for Asthma Complete Remission. Youden Index 72.5%. Abbreviations: ROC, Receiver operating characteristic; AUC, area under the curve.

To further evaluate the predictive power of lung function parameters, ROC curve analysis was conducted. The FEV1 percentage demonstrated an AUC of 0.900 (95% CI: 0.732–1.0, *p* = 0.024), indicating excellent discriminative ability in predicting complete remission. The optimal FEV1 cutoff value was determined to be 72.5%, which provided a sensitivity of 100% and a specificity of 70%. The FVC percentage also showed strong predictive accuracy with an AUC of 0.850 (95% CI: 0.639–1.0, *p* = 0.048) and an optimal cutoff of 91%, achieving the same sensitivity and specificity values (Figure 3).

### 4.5. Sensitivity and Specificity

The sensitivity and specificity analyses revealed that an FEV1 percentage cutoff of 72.5% provided a perfect sensitivity (100%) and a specificity of 70%, with a positive predictive value (PPV) of 57% and a negative predictive value (NPV) of 100%. Similarly, an FVC percentage cutoff of 91% yielded the same sensitivity and specificity, with identical PPV and NPV values. These metrics highlight the utility of these lung function parameters in clinical practice, particularly in predicting which patients with severe asthma and bronchiectasis are most likely to achieve clinical remission following biological therapy. The high NPV associated with both cutoffs suggests that patients with lower baseline lung function are less likely to achieve remission, emphasizing the need for targeted interventions in this group (Table 3).

Unpaired Student’s *t*-test or the Mann–Whitney test was used for the comparison of continuous parametric and nonparametric variables. Fisher exacts test was used for comparisons of categorical variables. Statistically significant *p*-values are highlighted in bold.

## 5. Discussion

To our knowledge, this is the first study to demonstrate, in a group of severe asthma (SA) patients with non-colonized bronchiectasis, that biological therapies targeting T2-high cells effectively achieve clinical remission in 28.6% of cases. This remission rate is comparable to that observed in SA patients without bronchiectasis. Moreover, we discovered that an FEV1 above 72.5% significantly predicts complete clinical remission, showing an exceptional sensitivity and negative predictive value (NPV) of 100%.

To better understand the impact of biological therapies on clinical remission, we intentionally excluded patients with microbiological colonization from our study. This approach helped us to clearly assess the therapies’ effects without the interference from neutrophilic flare-ups that often accompany colonization. Microbiological colonization can greatly exacerbate bronchiectasis by sustaining chronic inflammation and recurring infections, which not only intensifies respiratory symptoms but also increases the frequency of exacerbations. These complications make it challenging to manage patients who have both bronchiectasis and type 2-high asthma, as the colonization can provoke neutrophilic inflammation during exacerbations [3,4].

In non-cystic fibrosis bronchiectasis, approximately 27.1% of patients are colonized by Pseudomonas aeruginosa, the most prevalent pathogen, with others such as Haemophilus influenzae, Klebsiella pneumoniae, and Staphylococcus aureus also commonly found. Patients with microbial colonization tend to have poorer clinical outcomes, including higher rates of exacerbations, more severe symptoms, quicker deterioration of lung function, and increased hospitalizations. This colonization significantly influences treatment and prognosis, requiring more aggressive and specialized management strategies to address these negative impacts [23].

In our population, 28.6% of patients achieved clinical remission, aligning with the clinical remission rates observed in patients with pure severe asthma without bronchiectasis. The remission rates for severe asthma treated with different biological therapies vary, reflecting the complexity of these treatments. Specifically, therapies targeting IL-5/IL-5R, such as mepolizumab and benralizumab, show a clinical remission rate ranging from approximately 19% to 30% [24,25]. This is consistent across various studies, suggesting that nearly one-fifth to one-third of patients using these biologics can achieve significant control over their asthma symptoms and minimize exacerbations [26]. Dupilumab, an anti-IL-4Ra therapy, exhibits a higher remission rate, with about 30% of patients reaching remission [27], owing to its effective targeting of the IL-4/IL-13 pathway, crucial for type 2 inflammation-driven asthma [28]. On the other hand, anti-IgE therapies like omalizumab report a lower remission rate of about 6% [29], potentially due to IgE’s downstream role in the inflammatory cascade, which may render it less effective in achieving complete disease control across a broader patient base [30]. In real-world studies, the rates of clinical remission can vary slightly from clinical trial results, reflecting the broader and often more complex patient populations seen in routine clinical practice. For example, real-world data from the UK Severe Asthma Registry indicated that about 18% of patients achieved clinical remission after one year of biologic therapy, a figure slightly lower than those reported in controlled trials but still significant [31].

In various studies, clinical remission rates for patients with severe eosinophilic asthma (SEA) and bronchiectasis (BE) have varied, particularly when microbiological colonization and the Bronchiectasis Severity Index (BSI) are considered. Research, such as that by Campisi [7], indicates that remission is achievable but at lower rates among patients with microbiological colonization, and it decreases even further in those with higher BSI scores. Our study, however, observed a higher remission rate of 28.6% after 12 months on biologic therapies—significantly more than the 14.3% in Campisi et al.’s SEA + BE group, where 60% had severe BSI. This increase is likely due to our group of patients having mostly lower, mild-to-moderate BSI scores, a result of excluding colonized patients. Moreover, Mepolizumab showed increased effectiveness in patients with lower BSI scores [4], further supporting the observation that less severe bronchiectasis correlates with better treatment outcomes comparable to those of other severe asthma patients without bronchiectasis.

There are multiple factors that contribute to the success of therapies targeting T2-high inflammation in patients with severe asthma (SA) and bronchiectasis (BE). Research by Shoemark et al. [32] revealed that approximately 20% of individuals with BE, excluding those affected by asthma or allergic bronchopulmonary aspergillosis, show signs of eosinophilic inflammation. This highlights the variability of inflammatory responses in BE, especially among those with a T2-high phenotype. When SA coexists with BE, patients typically experience a combination of eosinophilic and neutrophilic inflammation, which plays a role in the remodeling of the airways. Eosinophils contribute to this process by releasing harmful proteins such as eosinophilic peroxidase and eosinophilic cationic protein, which damage the mucociliary epithelium, disrupt mucus clearance, and lead to the formation of mucus plugs [7]. Despite the mean eosinophil count being 465 cells/μL in our group, peripheral eosinophilia did not emerge as a significant predictor of clinical remission. One possible explanation is the varying effects of different biologic therapies on eosinophil counts due to their distinct mechanisms of action. Benralizumab induces rapid and near-complete depletion of eosinophils by targeting the IL-5 receptor and engaging natural killer cells, leading to apoptosis of eosinophils. Mepolizumab reduces eosinophil levels by blocking IL-5, which decreases eosinophil survival but does not eliminate them entirely. Omalizumab targets IgE and has an indirect effect on eosinophils, resulting in milder reductions. Dupilumab inhibits the IL-4 and IL-13 pathways, leading to variable effects on eosinophil counts [2].

This heterogeneity in treatment mechanisms within our patient group might have influenced the role of peripheral eosinophilia as a predictive marker for remission. Additionally, it is possible that many patients had mixed eosinophilic and neutrophilic inflammation. The presence of neutrophilic inflammation could diminish the effectiveness of therapies targeting eosinophils alone, thereby reducing the predictive value of peripheral eosinophil counts for clinical remission in this population.

Another important finding of our research is that a %FEV1 cut-off of 72.5% is a significant predictor for clinical remission in our population. We determined the FEV1 cutoff of 72.5% using ROC curve analysis, with the Youden Index identifying the optimal threshold. This value demonstrated 100% sensitivity and 70% specificity, indicating that patients with FEV1 above 72.5% are more likely to achieve clinical remission. Studies have shown that preserved lung function is a crucial determinant in the success of biological therapies, as patients with better lung function tend to have less irreversible airway remodeling and respond more favorably to treatment [33].

The introduction of a 72.5% FEV_1_ cutoff underscores its importance in predicting less extensive airway remodeling for patients who exceed this threshold, evidenced by its exceptional sensitivity and negative predictive value. This finding suggests that FEV_1_ could serve not only as a marker of disease severity but also as a predictive parameter for clinical remission, where higher values may indicate a better prognosis and a greater likelihood of achieving remission. This threshold is pivotal, as structural changes within the bronchial walls—typical of airway remodeling—play a significant role in exacerbating asthma and bronchiectasis, leading to persistent airflow limitation and decreased responsiveness to treatments [34]. Higher FEV_1_ values indicate a likelihood of milder airway remodeling, thereby enhancing responsiveness to biological therapies that effectively target inflammation [33]. Thus, FEV_1_ emerges as both a marker of disease severity and a predictive factor for clinical remission, suggesting that higher levels may predict a better prognosis and higher chances of achieving remission. Traditionally, FEV_1_ values below 80% of predicted have been used to indicate impaired lung function in asthma patients, as per ERS/ATS technical standards [35]. The more stringent FEV_1_ cutoff of 72.5% adopted in this study better identifies patients at a higher risk of adverse outcomes who might benefit most from aggressive biologic treatment, reflecting recent findings that lower FEV_1_ thresholds are indicative of more severe disease and obstruction in complex cases involving asthma and bronchiectasis [8]. This approach supports a nuanced understanding of how bronchiectasis and severe asthma interact, particularly in patients with a severe BSI, highlighting a complex and difficult-to-control form of the disease [36].

### 5.1. Implications for Clinical Practice

The results of this study have several implications for clinical practice. First, they highlight the need for careful patient selection and monitoring when prescribing biologic therapies to patients with severe asthma and bronchiectasis. Lung function tests should be routinely performed to identify patients most likely to benefit from these treatments. Additionally, our findings support the use of a composite endpoint, including clinical remission criteria, as a more comprehensive measure of treatment success beyond the traditional focus on exacerbation reduction alone.

Second, the study underscores the importance of addressing comorbidities like bronchiectasis in the management of severe asthma. While biologics offer significant benefits, the presence of bronchiectasis necessitates a more nuanced approach, potentially involving adjunctive therapies to manage infections and prevent further airway damage.

### 5.2. Limitations and Future Research

Despite these promising results, the study has several limitations. The relatively small sample size may limit the generalizability of the findings, particularly in a broader, more diverse patient population. Additionally, the exclusion of patients with chronic microbiological colonization means that the results may not fully reflect the challenges faced in typical clinical practice, where such colonization is common. Another limitation of our study is the lack of sputum cellular composition analysis, which could provide valuable insights into the predictive value of mixed inflammatory profiles for clinical remission. The short duration of follow-up also limits the ability to assess the long-term sustainability of clinical remission and the potential for relapse. Furthermore, while the study identified predictive parameters for clinical remission, the interplay between these factors, the extent of airway remodeling, and the BSI was not fully explored, warranting further investigation.

In conclusion, this study provides evidence that biological therapies can achieve clinical remission in a subset of patients with severe asthma and bronchiectasis characterized by T2-high inflammation. The adoption of a new FEV_1_ cutoff of 72.5% offers a more accurate assessment of patient risk and highlights the importance of lung function as a predictive marker for clinical remission. While FEV1 emerged as the strongest predictor, other factors, such as lung function parameters and inflammatory markers, may also play a role in clinical outcomes and should be further investigated in larger studies. Future research should focus on the long-term effects of biological therapies in this population, particularly in patients with chronic microbiological colonization, and further explore the role of airway remodeling and BSI in predicting treatment outcomes. Addressing these areas will be critical in refining treatment strategies and improving the quality of patients with severe asthma and bronchiectasis.

## Figures and Tables

**Table 1:** Baseline characteristics and clinical comparison of asthmatic patients with complete and incomplete remission.

	Population *N* = 14	Complete Remission *N* = 4	No Complete Remission *N* = 10	*p*
Age y m ± sd	58 ± 14.21	60.5 ± 13.17	57.0 ± 15.16	0.694
Sex F *n* (%)	5 (35.7)	1 (25)	4 (40)	0.545
BMI (Kg/m^2^) m ± sd	25.50 ± 3.81	27.0 ± 4.08	24.90 ± 3.75	0.373
Smoke *n* (%)				0.455
Yes	5 (35.7)	2 (50)	3 (30)	
Ex	0 (0)	0 (0)	0 (0)	
No	9 (64.3)	2 (50)	7 (70)	
Charlson Comorbidity Index Median (IQ 75)	2 (0–3)	3 (1–5)	3 (2.5–3)	0.825
Allergy yes *n* (%)	9 (64.3)	3 (75)	6 (60)	0.545
Eosinophilic Pneumonia yes *n* (%)	0 (0)	0 (0)	0 (0)	1
Hypereosinophilic Syndrome *n* (%)	0 (0)	0 (0)	0 (0)	1
Rhinosinusitis yes *n* (%)	6 (42.9)	2 (50)	6 (40)	0.594
Nasal Polyposis yes *n* (%)	5 (35.7)	3 (50)	3 (30)	0.455
Urticaria yes *n* (%)	0 (0)	0 (0)	0 (0)	1
Vocal Cord Dysfunction yes *n* (%)	0 (0)	0 (0)	0 (0)	1
GERD yes *n* (%)	4 (28.6)	1 (25)	3 (21.4)	0.689
OSAS yes *n* (%)	1 (7.1)	0 (0)	1 (10)	0.714
EGPA yes *n* (%)	0 (0)	0 (0)	0 (0)	1
Depressive Anxious Syndrome yes *n* (%)	1 (7.1)	1 (25)	0 (0)	0.286
Duration of Disease m ± sd	21.85 ± 17	12.0 ± 14.44	25.80 ± 16.95	0.180
Age of Diagnosis m ± sd	36.35 ± 23.11	48.50 ± 21.48	31.50 ± 22.93	0.227
T0 Number of Exacerbations *n* (%)	3 (2–5)	4 (3–5)	5 (5–5)	0.886
T0 Number of Visits to the ER *n* (%)	0 (0–1)	0.5 (0–1)	0.5 (0–1)	0.441
T0 Antibiotic Use Last Year y *n* (%)	1 (0–1)	0.5 (0–1)	0.5 (0–1)	0.327
T0 OCS Cycles year *n* (%)	0 (0–1)	1 (1–1)	0.5 (0–1)	0.990
T0 OCS Dose mg/dl *n* (%)	0 (0–5)	6.25 (0–12.5)	6.25 (0–12.5)	0.990
T0 LTRA yes *n* (%)	6 (42.9)	2 (50)	4 (40)	0.594
T0 ACT median (IQ 75)	19.5 (18–22)	19.5 (18–21)	14 (6–22)	0.799
T0 ACQ 6 median (IQ 75)	1.9 (1.6–4.5)	1.75 (1.45–2.87)	2.60 (1.52–5.23)	0.395
T0 TAI *n* (%)	54 (53–54)	54 (49–54)	54 (53–54)	0.543
T0 %FEV1 m ± sd	72.85 ± 24.42	92.25 ± 15.64	65.10 ± 23.36	0.034
T0 FEV1 l m ± sd	1.94 ± 0.75	2.21 ± 0.78	1.83 ± 0.75	0.443
T0 %FVC_ m ± sd	86.14 ± 18.94	101.75 ± 8.18	79.90 ± 18.56	0.010
T0 FVC_l m ± sd	2.78 ± 0.80	2.96 ± 0.82	2.71 ± 0.83	0.632
T0 FEV1/FVC m ± sd	81.21 ± 15.88	88.25 ± 17.15	78.40 ± 15.34	0.313
T0 FeNO50 (ppb) m ± sd	19.92 ± 9.71	19.25 ± 12.63	20.20 ±9.10	0.876
T0 WBC m ± sd	7882.12 ± 1663.75	8672.5 ± 2140.8	7566 ± 1442.86	0.278
T0 % eos m ± sd	5.94 ± 2.90	4.24 ± 1.15	6.63 ± 3.15	0.174
T0_eos (n/µL) m ± sd	465.21 ± 252.92	375.5 ± 158.28	501.10 ± 281.14	0.423
T0 IgE kUA/L m ± sd	258.88 ± 566.84	120.35 ± 91.04	314.30 ± 670.38	0.584
T0 BSI Median (IQ 75)	6.5 (4–8)	5.5 (2–7.5)	7 (4–8)	0.669
T0 Laterality Bronchiectasis Median (IQ 75)	10 (71.4)	2 (50)	8 (51.7)	0.311
Type Of Bronchiectasis *n* (%)				0.627
cylindrical	6 (42.9)	1 (25)	5 (50)	
varicose	6 (42.9)	2 (50)	4 (40)	
cystic	2 (14.3)	1 (25)	1 (10)	
Grading BSI *n* (%)				0.689
mild	4 (28.6)	1 (25)	3 (30)	
moderate	10 (71.4)	3 (75)	7 (70)	
severe	0(0)	0 (0)	0 (0)	
T0_FACED score	1 (0–2)	1 (0–2)	2 (1–3)	0.157
T1 AdversEvent *n* (%)	0 (0)	0 (0)	0 (0)	1
T1 Number of Exacerbation *n* (%)	1 (0–1)	0 (0–0)	1 (0–1)	0.023
T1 Number of Visits To the Emergency Room *n* (%)	0 (0–0)	0 (0–0)	0 (0–0)	1
T1 Antibiotic Use Last Year *n* (%)	0 (0–1)	0 (0–0)	0 (0–1)	0.053
T1 RELIVER las month yes *n* (%)	0 (0–0)	0 (0–0)	0 (0–0)	0.527
T1 OCS cycles last month *n* (%)	0 (0–0)	0 (0–0)	0 (0–0)	0.352
T1 Mean cycle dose last month mg/dl median (IQ 75)	0 (0–0)	0 (0–0)	0 (0–0)	0.352
Biological Therapy *n*.				0.241
Omalizumab	3	0	3	
Mepolizumab	2	1	1	
Benralizumab	6	1	5	
Dupilumab	3	2	1	
T1_LTRA *n* (%)	5 (35.7)	1 (25)	4 (40)	0.545
T1_ACT median (IQ 75)	19.5 (15–23)	22 (20–24)	18 (15–20)	0.064
T1_ACQ6 median (IQ 75)	1.6 (0.6–3.5)	0.7 (0.52–0.95)	2.5 (1.02–4.25)	0.065
T1_TAI median (IQ 75)	54 (54–54)	54 (54–54)	54 (53–54)	0.826
T1 %FEV1 m ± sd	75.71 ± 30.65	96.25 ± 9.87	67.5 ± 32.59	0.028
T1 FEV1 l m ± sd	1.98 ± 0.94	2.29 ± 0.79	1.18 ± 1.00	0.457
T1 %FVC m ± sd	84.61 ± 28.46	107.75 ± 18.06	74.33 ± 26.58	0.028
T1 FVC l m ± sd	2.64 ± 1.10	3.12 ± 1.07	2.43 ± 1.10	0.330
T1_FEV1/FVC m ± sd	83.46 ± 16.59	89.75 ± 13.27	80.66 ± 17.83	0.385
T1 FeNO50 (ppb) m ± sd	16.64 ± 12.38	17 ± 0.81	16.5 ± 14.87	0.310
T1 WBC (n/µL) m ± sd	6524 ± 1126.6	6505 ± 1675.44	6531.6 ± 947.47	0.556
T1 %eos m ± sd	1.18 ± 0.71	1.07 ± 0.79	1.22 ± 0.71	0.745
T1 eos (n/µL) m ± sd	77.98 ± 60.03	85.25 ± 72.99	75.07 ± 58.28	0.787
OCS yes median (IQ 75)	4 (28.6)	(0)	4 (40)	0.210
EXACERBATION yes *n* (%) median (IQ 75)	7 (50)	0 (0)	7 (70)	0.035
DELTA FEV1 l m ± sd	−0.72	0.08 ± 0.33	0.02 ± 0.52	0.852
DELTA FVC l m ± sd	−0.90	0.165 ± 0.375	−0.16 ± 0.43	0.206

Abbreviations: y = years; F = female; ER = emergency Room; BMI, Body Mass Index; GERD, gastro-esophageal reflux; BSI, Bronchiectasis Severity Index; ACT, Asthma Control Test; OCS, oral corticosteroids (prednisone); FEV1, forced expiratory volume in the 1st second; FVC, forced vital capacity; IgE, immunoglobulin-E; FeNO, Fractional Exhaled Nitric Oxide; m ± sd = Mean ± Standard Deviation; IQ 75: interquartile 75%; ppb, part per bilion.

**Table 2:** Univariate logistic regression analysis for predictors of clinical remission.

	Univariate Regression		
	ODD	CI 95%	*p*
Age y	1.020	0.932–1.117	0.668
Sex F	0.500	0.037–6.683	0.600
BMI Kg/m^2^	1.163	0.847–1.598	0.351
Smoke Yes	2.333	0.216–25.43	0.486
Charlson Comorbidity Index	1.046	0.480–2.282	1.046
Rhinosinusitis y	1.500	0.146–15.461	0.733
Nasal_polyposis y	2.333	0.216–25.245	0.486
GERD y	0.778	0.056–10.861	0.852
Duration of Disease y	0.937	0.851–1.033	0.191
Age of Diagnosis y	1.037	0.979–1.099	0.218
Allergy	2.000	0.150–26.734	0.600
T0 Number of Exacerbations	0.944	0.583–1.528	0.813
T0 Number of Visit ToTheEmergency Room	0.333	0.025–4.401	0.404
T0 Antibiotic Use Last Year	1.287	0.571–2.905	0.543
OCS_cycles_year	5.676	0.552–58.335	0.144
OCS Dose mg	5.676	0.552–58.355	0.144
T0 LTRA	1.500	0.146–15.461	0.733
T0 ACT	1.052	0.809–1.367	0.707
T0 ACQ 6	0.579	0.219–1.533	0.271
T0 TAI	0.846	0.485–1.474	0.846
T0 %FEV1_	1.008	1.000–1.167	0.050
T0 FEV1_l	2.079	0.395–10.942	0.368
T0 %FVC	1.118	0.978–1.278	0.103
T0 FVC_l	1.513	0.330–6.937	0.594
T0 FEV1/FVC	1.046	0.962–1.137	0.294
T0 FeNO50ppb	0.989	0.874–1.120	0.864
T0 WBC n/µL	1.000	1.000–1.001	0.263
T0 %eos	0.604	0.281–1.301	0.198
T0 eos n/µL	0.997	0.991–1.004	0.404
T0 IgE kUA/L	0.999	0.993–1.004	0.648
T0 BSI	0.840	0.519–1.361	0.479
T0 FACED score	0.431	0.121–1.540	0.195
T0 Laterality Bronchiectasis	0.250	0.021–3.041	0.277
Type of Bronchiectasis	2.269	0.409–12.590	0.349
Grading BSI	1.286	0.092–17.954	0.852
BiologicalTherapy	2.678	0.584–12.285	0.205

Abbreviations: y = years; F = female; BMI, Body Mass Index; GERD, gastro-esophageal reflux; BSI, Bronchiectasis Severity Index; ACT, Asthma Control Test; OCS, oral corticosteroids (prednisone); FEV1, forced expiratory volume in the 1st second; FVC, forced vital capacity; IgE, immunoglobulin-E; FeNO, Fractional Exhaled Nitric Oxide; FACED, forced expiratory volume in 1 s (FEV1), Age, chronic colonization, extension, and dyspnea.

**Table 3:** Sensitivity, specificity, PPV, NPV for clinical remission based on FEV1 and FVC cutoffs.

	Sensitivity	Specificity	PPV	NPV
%FEV1 cut off 72.5	100	70	57	100
%FVC cut off 91	100	70	57	100

Abbreviations: FEV1, forced expiratory volume in the 1st second; FVC, forced vital capacity; PPV, positive predictive value; NPV, negative predictive value.

## Data Availability

Due to privacy and ethical considerations, I can provide the dataset supporting the reported results upon request. Interested parties can contact me directly via email to request access.
